# Targeting Wnt/β-catenin activation in combination with temozolomide leads to glioblastoma inhibition and long-term survival in mice

**DOI:** 10.1016/j.gendis.2025.101624

**Published:** 2025-04-04

**Authors:** Xiaowei Zhang, Zhongyong Wang, Taohui Ouyang, Brayden Wang, Richard I. Joh, Suyun Huang

**Affiliations:** a Department of Human and Molecular Genetics, School of Medicine, Virginia Commonwealth University, Richmond, VA 23298, USA; b Massey Cancer Center, Virginia Commonwealth University, Richmond, VA 23298, USA; c Department of Pharmacology and Toxicology, Virginia Commonwealth University, Richmond, VA 23298, USA; d Department of Physics, Virginia Commonwealth University, Richmond, VA 23219, USA; e Department of Neurosurgery, The University of Texas MD Anderson Cancer Center, Houston, TX 77030, USA

**Keywords:** Chemoresistance, Glioblastoma, Temozolomide, Wnt inhibitor, Wnt/β-catenin

## Abstract

Glioblastoma (GBM) is one of the most treatment-resistant brain malignancies. Dysregulation of Wnt/β-catenin signaling might be implicated in tumorigenesis of GBM. In this study, spatial transcriptomic analysis revealed elevated expression of Wnt target genes within tumor regions compared with peritumoral tissues. Overexpression of porcupine (PORCN), an O-acyltransferase for Wnt secretion, correlated with poor prognosis of GBM patients. Treatment with Wnt-C59, a PORCN inhibitor, inhibited Wnt signaling, hindered GBM cell proliferation and invasion, and inhibited GBM stem cells' self-renewal properties in a dose-dependent manner. Moreover, using β-catenin knockout and knockdown cells, FOXM1 was identified as a downstream transcription target of Wnt/β-catenin signaling. Wnt-C59 inhibited the expression of FOXM1 in GBM cells. Furthermore, Wnt-C59 demonstrated the ability to impede tumor formation and enhance the overall survival of mice bearing GBM in a preclinical model. Notably, the combined treatment of Wnt-C59 with temozolomide further enhanced therapeutic outcomes, leading to a significant extension of overall survival in GBM-bearing mice. Mechanistically, Wnt-C59 significantly down-regulated the expression of oncogenic targets associated with Wnt/β-catenin signaling (FOXM1, cyclin D, and C-Myc) both *in vitro* and in orthotopic GBM models. Our findings reveal that targeting Wnt/β-catenin signaling via PORCN inhibition, especially in combination with temozolomide, offers a promising therapeutic strategy for treating GBM.

## Introduction

Malignant gliomas, particularly glioblastomas (GBMs), represent the most aggressive form of brain tumors and are currently incurable. Despite maximal surgical resection, chemotherapy, and radiation, the lethality of GBM is inevitable due to tumor recurrence.^1^ The molecular mechanisms underlying glioma development and progression are still poorly understood and more effective therapies are urgently needed.

The Wnt/β-catenin signaling pathway plays a critical role in development, differentiation, and cell proliferation.^2^^,^^3^ Wnt proteins interact with Frizzled receptors and the low-density lipoprotein receptor-related protein 5/6 (LRP5/6) co-receptor, which in turn activates Dishevelled. This activated Dishevelled prevents the destruction of β-catenin, allowing it to stabilize. Once stabilized, β-catenin builds up in the cytoplasm and moves into the nucleus. Inside the nucleus, β-catenin binds with TCF/LEF transcription factors, initiating the transcription of Wnt target genes. Growing evidence supports the role of the Wnt/β-catenin pathway in tumorigenesis, including glioma. Current literature has linked this signaling pathway with multiple facets of gliomagenesis, including cell proliferation and invasion.^4^, ^5^, ^6^, ^7^ Moreover, our previous studies have shown that Wnt/β-catenin signaling is required for the self-renewal and tumor-initiating abilities of GBM stem cells (GSCs).^4^^,^^5^ Wnt signaling stabilized the forkhead box M1 (FOXM1) protein, a transcription factor belonging to the forkhead box (Fox) family. FOXM1 regulated the nuclear localization of β-catenin and promoted the tumorigenesis of GBM.^4^ The Cancer Genome Atlas (TCGA) gene expression data indicate that FOXM1 is markedly overexpressed in GBM clinical samples relative to non-tumor tissues.^8^ Additionally, FOXM1 expression in human glioma tissue correlates directly with glioma grade, and its level in human GBM tissue inversely correlates with patient survival.^9^ FOXM1 has also been reported to contribute to temozolomide (TMZ) resistance through its interaction with Rad51.^10^ These data suggest that targeting the Wnt/β-catenin signaling pathway might be an effective therapeutic approach for GBM.

Porcupine enzyme (PORCN) is membrane bound O-acyltransferase family that is essential for Wnt protein palmitoylation. This modification is crucial for the secretion of Wnt proteins, indicating that PORCN is a key modulator of Wnt signaling.^11^^,^^12^ Wnt-C59 is a small molecule inhibitor of PORCN, which prevents the palmitoylation of Wnt proteins, thereby blocking their secretion and subsequent signaling pathways.^13^ This compound has previously been reported to exert anti-tumor activity against mammary tumors without toxicity in MMTV-WNT1 transgenic mice.^14^ However, the effects of Wnt-C59 on gliomas, particularly through Wnt/β-catenin pathway inhibition, have not been explored. The current study evaluated the efficacy of Wnt-C59 in reducing GBM tumorigenicity both *in vitro* and *in vivo.* Additionally, the combined effect of Wnt-C59 with TMZ was investigated using a GBM xenograft model.

## Materials and methods

### Cell lines and culture conditions

NHA, U87MG, and LN229 cell lines were cultured in a CO_2_ incubator at 37 °C in Dulbecco's modified Eagle's medium (DMEM) supplemented with 10% bovine calf serum (HyClone). GSCs (GSC11, GSC17, GSC20, and GSC23) were obtained from fresh surgical glioblastoma specimens and cultured in DMEM/F-12 medium supplemented with heregulin B27, epidermal growth factor (10 ng/mL), and basic fibroblast growth factor (10 ng/mL). The characteristics of the GSCs were presented previously.^15^ Additionally, *β-catenin*^*+/+*^ and *β-catenin*^*−/−*^ mouse embryonic fibroblasts (MEFs) were kindly provided by Dr. Xi He (Boston Children's Hospital, Boston, Massachusetts, USA) and were cultured as described previously.^16^

### Western blotting analysis

GSC11 and U87MG cells were treated with various concentrations of Wnt-C59 for 48 h. After treatment, cells were collected and homogenized using RIPA lysis buffer. The resultant total proteins were subsequently fractionated into 8%–12% SDS-PAGE gels and then transferred onto polyvinylidene difluoride membranes (Millipore). Following blocking with 5% nonfat dry milk in tris-buffered saline solution containing 0.1% Tween 20, the membranes were subjected to overnight incubation with primary antibodies, followed by incubation with horseradish peroxidase-conjugated secondary antibodies (Santa Cruz Biotechnology). At last, protein bands were visualized using an enhanced chemiluminescence substrate. The antibodies used in this study include phosphorylated LRP6 (LRP6-phospho-S1490 antibody, Abcam), FOXM1, cyclin D1, c-Myc, β-catenin, Rad51 (Cell Signaling Technology), Sox2 (Abcam), and Nestin (BD Transduction Laboratories).

### Immunohistochemical analysis

Tissue sections from GBM xenografts were prepared for immunohistochemical staining by deparaffinization and rehydration with an ethanol series. Antigen retrieval was then performed using sodium citrate or tris-EDTA buffer according to the antibody manufacturer's instructions. Sections were immersed in 3% H_2_O_2_ solution in phosphate buffer saline at room temperature for 15 min to block endogenous peroxidases and were then blocked with 3% bovine serum albumin in tris-buffered saline solution at room temperature for 60 min. The sections were incubated overnight with primary antibody at 4 °C, followed by immunohistochemical staining using horseradish peroxidase-conjugated secondary antibodies and diaminobenzidine as the chromogen.

### Immunofluorescence cell staining

Cells were cultured on chamber slides pre-coated with poly-l-ornithine and fibronectin. Cells were then fixed with 4% paraformaldehyde, permeabilized for 5 min with phosphate buffer saline solution containing 0.1% Triton X-100 (PBS-T), and then quenched with 50 mM NH_4_Cl in PBS-T. Blocking was performed using 1% bovine serum albumin in PBS-T. Sections were immunostained with FOXM1 and β-catenin antibodies, followed by staining with DAPI and anti-rabbit IgG conjugated with Alexa 488 (Thermo Fisher Scientific). The fluorophore was excited using lasers at 405 and 488 wavelengths, and images were captured with a confocal microscope (Olympus FluoView FV1000).

### MTT assay

The MTT assay was conducted to assess cell viability. U87MG cells (2 × 10^3^) and GSC11 cells (5 × 10^3^) were seeded into 96-well plates. At specified time points, 20 μL of MTT solution (5 mg/mL) was added to each well, followed by a 4-h incubation at 37 °C. Subsequently, 100 μL of dimethyl sulfoxide (DMSO) was added to each well to lyse the cells. Absorbance was then recorded at 570 nm using a microplate reader.

### Colony formation assay

Cells (2 × 10^3^) were suspended in culture media with 0.3% (wt/vol) agarose, supplemented with either Wnt-C59 (1 μM) or DMSO as a vehicle control. The cell suspension was then layered onto 0.6% (wt/vol) agarose in 24-well plates. Subsequently, the plates were kept at 37 °C for 14 days. Colonies formed were subsequently stained with p-iodonitrotetrazolium violet (1 mg/mL) and counted.

### Neurosphere formation assay

GSC11 and U87MG cells were seeded individually in 96-well plates at a density of 1 cell/μL. These cells were treated with either vehicle (control), TMZ (0 μM or 25 μM), or various concentrations of Wnt-C59 (ranging from 0 μM to 5 μM). Following 7 days, cell growth and sphere formation were assessed. In addition, representative images were captured, and the resulting neurospheres were recorded for subsequent statistical analysis.

### Spatial transcriptomics data analysis

We downloaded spatial transcriptomics sequencing data from published studies^17^^,^^18^ (GBM#1 represents UKF269_T, GBM#2 represents UKF334_T, and GBM#3 represents patient 4). Using the “initiateSpataObject_10X” function, we created a “spata2“ object and conducted several analyses, including normalization, dimensionality reduction, and clustering. Wnt target genes were acquired from a previous study.^19^ We evaluated the mean expression levels of genes in WNT target genes in both tumor and adjacent non-tumor tissues. The specific computational code used in this analysis is available at https://themilolab.github.io/SPATA2/index.html.

### TCGA database analysis

We obtained RNA sequencing data in TPM format, uniformly processed by the Toil pipeline,^20^ from UCSC XENA (https://xenabrowser.net/datapages/). The data included samples from The Cancer Genome Atlas (TCGA) for glioblastoma multiforme and corresponding normal brain tissue from the Genotype-Tissue Expression (GTEx) project. Gene expression values were transformed using log_2_ (TPM + 1). For optimal grouping, the “surv_cutpoint” function from the “survminer” package was used to determine the best cut-off point.

### Quantitative real-time PCR

Total RNA was extracted using Trizol reagent (Invitrogen) and then was reverse-transcribed using the iScript™ Reverse Transcription Supermix (Bio-Rad) according to the manufacturer's instructions. The synthesized cDNAs were then amplified for quantitative PCR using SYBR Master Mix (Life Technologies). The primers were as follows: *FOXM1:* forward, 5′-AAGGTTGAGGAGCCTTCGAG-3'; reverse, 5′-ATTCGGTCGTTTCTGCTGCTT-3'; *GAPDH*, forward, 5′-CGGATTTGGTCGTATTGG-3'; reverse, 5′-TCCTGGAAGATGGTGATG-3'. All quantitative PCR results were from three independent assays.

### Luciferase reporter assay

Cells were transfected with TOP-Flash or FOP-Flash luciferase reporter constructs following the protocol described in our previous study.^4^ The transfection efficiency was standardized against a concurrently transfected TK-RL reporter, which expresses Renilla luciferase. Subsequently, luciferase activities were quantified utilizing a dual-luciferase reporter assay system (Promega).

### Tumor cell invasion assay

GSC11 and U87MG cells were seeded on 6-well plates and treated overnight with increasing concentrations of Wnt-C59. The invasion of GSC11 and U87MG cells was assessed using Matrigel® Invasion Chambers equipped with an 8-μm pore size polycarbonate filter. GSC11 or U87MG (3 × 10^5^ cells/mL) cell suspensions in 200 μL of medium (containing different concentrations of Wnt-C59) were added to the upper chamber, and 0.5 mL of complete medium containing 10% fetal bovine serum was added to the lower chamber. Following an 18-h incubation at 37 °C, the inserts were fixed with methanol for 20 min and stained with 0.1% (w/v) crystal violet. Then, non-invading cells were carefully removed from the upper chamber using cotton swabs. The cells on the bottom of membrane were photographed using an inverted microscope (200 × magnification). The experiments were performed in triplicates.

### Chromatin immunoprecipitation assay

After treatment with siRNA-control or β-catenin-siRNA for 48 h, U87MG cells (2 × 10^6^) were subjected to chromatin immunoprecipitation using the ChIP Assay Kit from Cell Signaling Technology, adhering strictly to the manufacturer's instructions. Chromatin was precipitated, and the DNA extracted was used to amplify a 317 bp region of the FOXM1 promoter with these following primers: 5′-CAAAAGACAGGTTTCGCGCTGAG-3' (forward) and 5′-CGAGGGAGAGTTTGGGGACG-3' (reverse). The PCR products were resolved electrophoretically on a 2% agarose gel and visualized using ethidium bromide staining.

### Intracranial human glioma xenograft model

All mouse experiments were approved by the Institutional Animal Care and Use Committee of MD Anderson Cancer Center. U87MG cells (5 × 10^5^) or GSC11 cells (1 × 10^5^) were injected intracranially into 6- to 8-week-old nude (nu/nu) mice as previously described.^15^ Symptomatic animals were euthanized, while the remaining animals were euthanized 90 days post-injection. Mouse brains were harvested, fixed in 4% formaldehyde, and paraffin-embedded. Brain tumor formation was determined by histological analysis of hematoxylin and eosin-stained brain tissue sections. The tumor diameter was measured. Therapeutic interventions began one day after cell implantation, with mice receiving either oral Wnt-C59 (10 mg/kg/day) or intraperitoneal TMZ (20 mg/kg/day) dissolved in a 1:4 ratio of dimethyl sulfoxide to polyethylene glycol 300 (Sigma). Treatments were administered every other day for 30 days. For combinatorial treatment, mice received injections of TMZ or oral Wnt-C59 on alternate days for 30 days. The vehicle alone was used for the negative control. Survival was monitored, and animals were euthanized either upon becoming moribund or at the 90-day endpoint. All experimental mice were housed in laminar flow cabinets under pathogen-free conditions and in accordance with institutional regulations, within facilities approved by the Association for Assessment and Accreditation of Laboratory Animal Care.

### Statistical analysis

Data are presented as mean ± standard error of the mean or standard deviation. To evaluate statistical differences between two groups, we performed a two-tailed student's *t*-test and two-sided Mann–Whitney test using R (version 4.3.0). Survival analysis was conducted using the Kaplan–Meier model with a two-sided log-rank test. A significance level of *p* < 0.05 was considered statistically significant.

## Results

### Wnt signaling pathway is highly activated in human GBM

**Figure 1:**
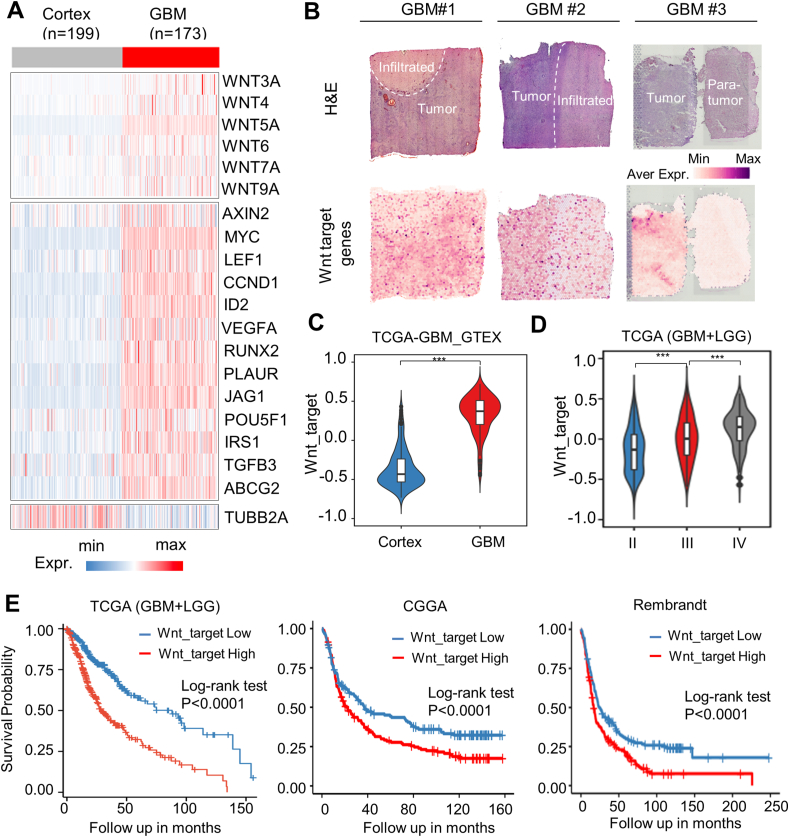
Overexpression of Wnt target genes positively correlates with glioma grade and predicts worse patient survival. **(A)** The heatmap showing the differential expression of the Wnt proteins and the Wnt target genes between the GTEx cortex (*n* = 199) and TCGA-GBM (*n* = 173) datasets. **(B)** The spatial transcriptomic data showed the expression distribution of Wnt target genes in GBM tissues. The samples include GBM1 and GBM2 (UKF269_T and UKF334_T; Vidhya, 2022), and GBM3 (Patient 4; Yan, 2023). **(C, D)** The violin plot displaying the GSVA scores of Wnt target genes across RNA sequencing data from GTEx brain, TCGA_LGG, and TCGA-GBM datasets. ∗∗∗*p* < 0.001. **(E)** Analyses of TCGA, CGGA, and Rembrandt datasets revealed that GSVA scores of Wnt target genes inversely correlated with glioma patient survival. The Wnt_target high- and low-expression groups were stratified based on the median GSVA score within each dataset.

We extracted data from GTEx cortex and TCGA-GBM databases and discovered that several Wnt proteins, including WNT3A, WNT4, WNT5A, WNT6, WNT7A, and WNT9A, were up-regulated in GBM compared with the normal cerebral cortex (Fig. 1A, up panel). Additionally, the Wnt target genes AXIN2, MYC, LEF1, CCND1, ID2, VEGFA, RUNX2, PLAUR, JAG1, POU5F1, IRS1, TGFB3, and ABCG2 also exhibited increased expression in GBM as compared with the normal cerebral cortex (Fig. 1A, bottom panel). We then conducted spatial transcriptomic analysis on tissues from three different GBM samples, revealing that the expression levels of the Wnt target genes were significantly higher in the tumor core compared with adjacent non-tumorous areas (Fig. 1B). Moreover, Wnt target gene expression was elevated in GBM compared with normal tissue, further increased as glioma progressed from lower-grade glioma to GBM (Fig. 1C, D), and was predictive of poor prognosis for glioma patients across multiple datasets (Fig. 1E).

### Wnt-C59 inhibits Wnt signaling in GBM cells

**Figure 2:**
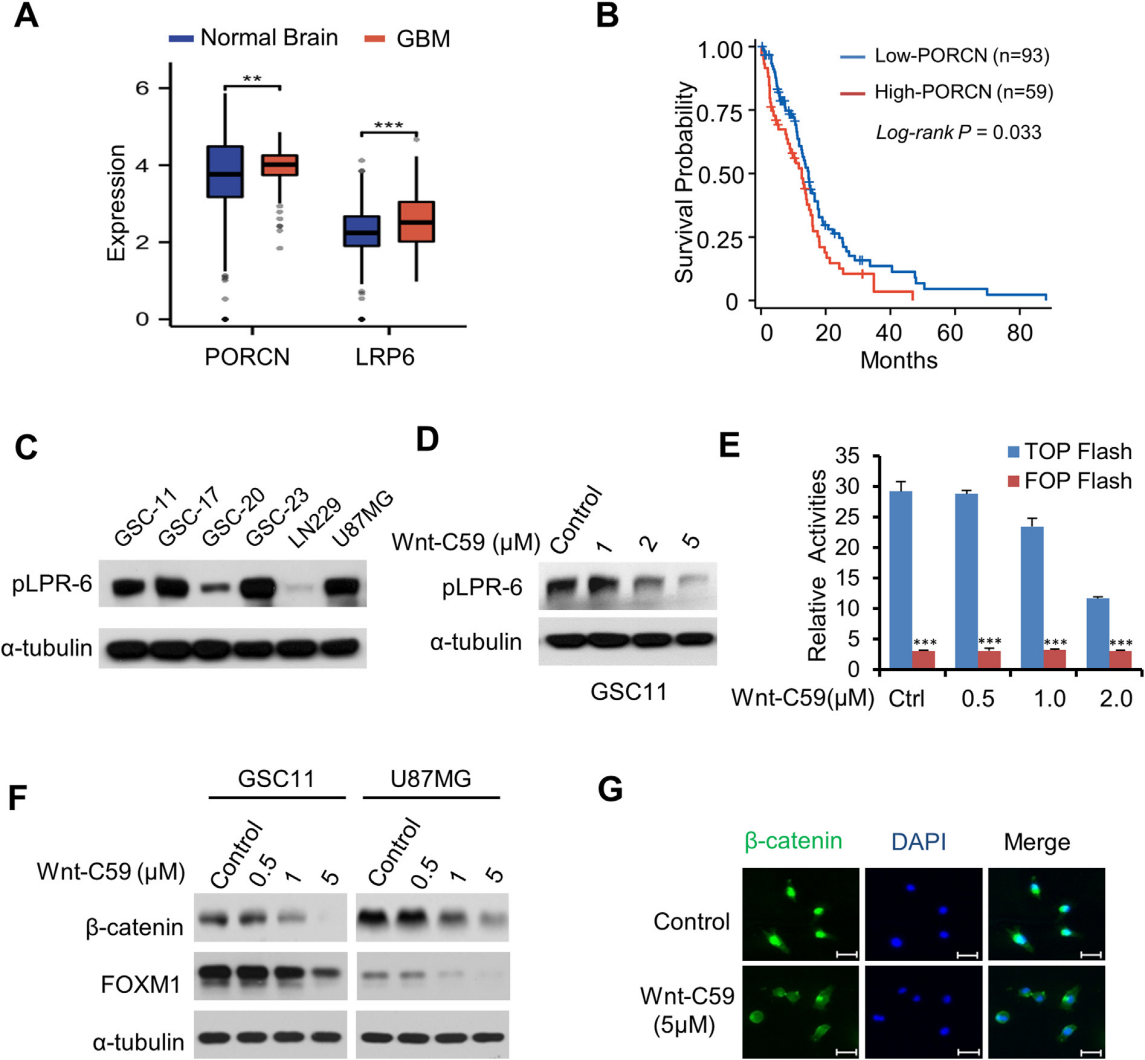
Wnt-C59 inhibits Wnt/β-catenin signaling in glioblastoma (GBM). **(A)** The expression levels of low-density lipoprotein receptor protein 6 (LRP6) and porcupine (PORCN) in GBM and normal brain tissues based on TCGA and GTEx databases. ∗∗∗*p* < 0.001. **(B)** Survival analysis of PORCN based on TCGA-GBM dataset. **(C)** Expression of pLPR6 in six cell lines including two GBM cell lines and four established GSC lines demonstrated with western blotting. **(D)** Expression of pLPR6 in GSC11 cells treated with increasing doses of Wnt-C59 were analyzed by western blotting analysis. **(E)** GSC11 cells were transfected with the TOP-Flash or control FOP-Flash reporter to determine reporter activities 48 h after Wnt-C59 treatment. TOP-Flash reporter activation (fold) was calculated relative to that of FOP-Flash. The values were mean ± standard deviation for triplicate samples. ∗∗∗*p* < 0.001. **(F)** Expression levels of β-catenin and forkhead box M1 (FOXM1) proteins in GSC11 cells and U87MG cells treated with increasing doses of Wnt-C59 were analyzed by western blotting analysis. **(G)** U87MG cells were treated with control DMSO or Wnt-C59 (5 μM) for 24 h. Cytoplasmic and nuclear levels of β-catenin in the cells were analyzed by double immunofluorescence staining for β-catenin and nuclei (DAPI, blue) (scale bar, 25 μM).

PORCN is a membrane bound O-acyltransferase family that is essential for Wnt palmitoylation and secretion, highlighting PORCN as a critical modulator of Wnt signaling. The analysis of the TCGA-GBM dataset revealed that PORCN was up-regulated in GBM tissues as compared with normal brain tissues, and its high expression was associated with poor prognosis in GBM patients (Fig. 2A, B). Furthermore, low density lipoprotein receptor-related protein 6 (LPR6) is one of the receptors of Wnt ligands. Upon ligation by Wnt, LRP6 is phosphorylated, initiating a transduction cascade that leads to β-catenin stabilization.^21^, ^22^, ^23^ Accumulated β-catenin then translocated into the nucleus and formed a transcriptional complex with TCF to activate the expression of Wnt-target genes.^24^ We found that LRP6 was highly phosphorylated (p-LRP6) in the majority of GBM cells, especially in GSCs (Fig. 2C). Taken together, these findings suggest that the Wnt signaling pathway plays an active role in human GBM, as evidenced by the up-regulation of Wnt ligands, the activated Wnt receptor, and Wnt target genes.

Based on the above findings, we hypothesized that treatment with Wnt-C59 would inhibit Wnt signaling activation in GBM cells. Wnt-C59 treatment reduced the phosphorylation of LRP6 in a dose-dependent manner in GSC11 cells (Fig. 2D). Wnt-C59 reduced Wnt activity in GSC11 cells in a dose-dependent manner, as measured by the TOP-Flash reporter assay (Fig. 2E), further supporting the observation that Wnt-C59 inhibits Wnt signaling pathway activation. Moreover, the protein levels of β-catenin decreased in a dose-dependent manner with Wnt-C59 treatment (Fig. 2F). Furthermore, the level of nuclear β-catenin, which indicates activated β-catenin, decreased following treatment with Wnt-59 in U87MG cells (Fig. 2G).

### Wnt-C59 inhibits growth and invasion of GBM cells and the self-renewal of GSCs

**Figure 3:**
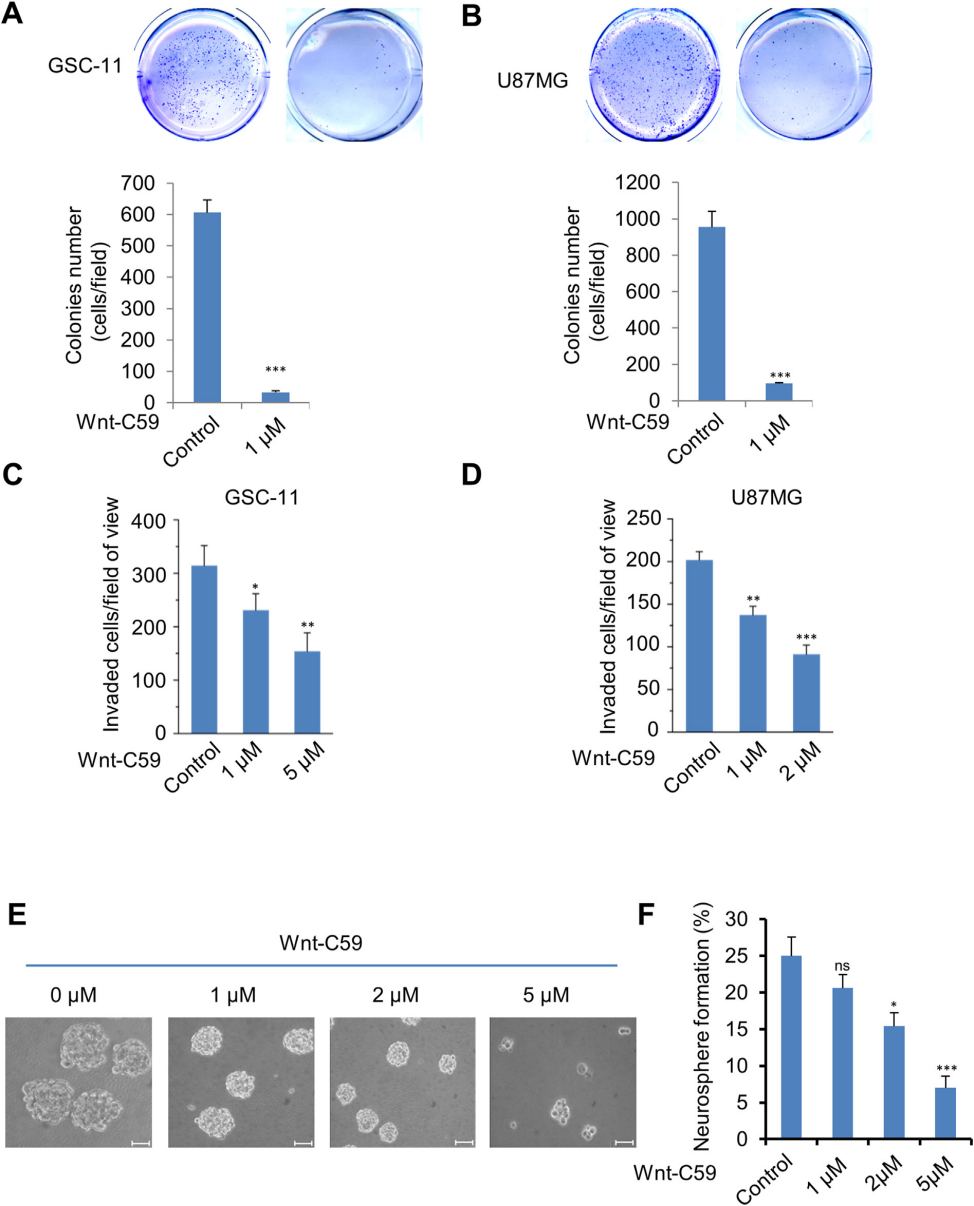
Wnt-C59 inhibits tumorgenicity of glioblastoma. **(A, B)** GSC11 (A) and U87MG (B) cells were treated with DMSO (control) or Wnt-C59 (1 μM) and growth was assessed with soft agar colony formation assay. Representative images are shown with and without Wnt-C59 treatment. The bar charts display the mean of the colony numbers of triplicate plates from a representative experiment (performed twice); Bars, standard error of the mean. ∗∗∗*p* < 0.001 as determined using Student's *t*-test. **(C, D)** GSC11 (C) and U87MG (D) cells treated with serial doses of Wnt-C59 or control were subjected to transwell invasion assays. The data were presented as mean ± standard error of the mean. ∗*p* < 0.05, ∗∗*p* < 0.01, and ∗∗∗*p* < 0.001. **(E)** Representative images of neurospheres of GSC11 cells after Wnt-C59 treatment. Scale bar, 50 μM. **(F)** The neurosphere formation efficiency was assessed in GSC11 cells. The formation assay was performed by plating dissociated single cells at a density of 1 cell/μL and counting the number of spheres that formed after 7 days. The values were mean ± standard deviation for triplicate samples. ∗*p* < 0.05 and ∗∗∗*p* < 0.001.

Previous studies indicate that Wnt signaling plays a crucial role in regulating the growth of GBM cells, likely through the activation of β-catenin. β-catenin drives GBM cell growth and invasion by promoting the transcriptional activation of proto-oncogenes such as cyclin D1, c-Myc, MMP-2, and uPAR, all of which are essential for the proliferation and invasion of GBM cells. To explore the impact of Wnt-C59 on GBM cell growth and invasion, we conducted a colony formation assay to assess anchorage-independent cell growth. Treatment with Wnt-C59 resulted in a significant reduction in the anchorage-independent growth of both U87MG and GSC11 cells (Fig. 3A, B). Importantly, Wnt-C59 did not show inhibition of colony formation in normal human astrocytes (NHA) compared with its effect on tumor cells (Fig. S1). We then evaluated cell invasiveness using the Matrigel invasion assay, where Wnt-C59 treatment led to a marked decrease in the invasion of both U87MG and GSC11 cells (Fig. 3C, D). Additionally, prior research indicates that Wnt signaling is involved in maintaining the stemness of GSC cells by influencing their proliferation and self-renewal. Therefore, we investigated the effects of Wnt-C59 on the stemness of GSC11 cells and found that Wnt-C59 treatment significantly reduced both the size and number of tumor spheres in a dose-dependent manner (Fig. 3E, F).

### β-catenin interacts with the FOXM1 promoter and regulates its expression

**Figure 4:**
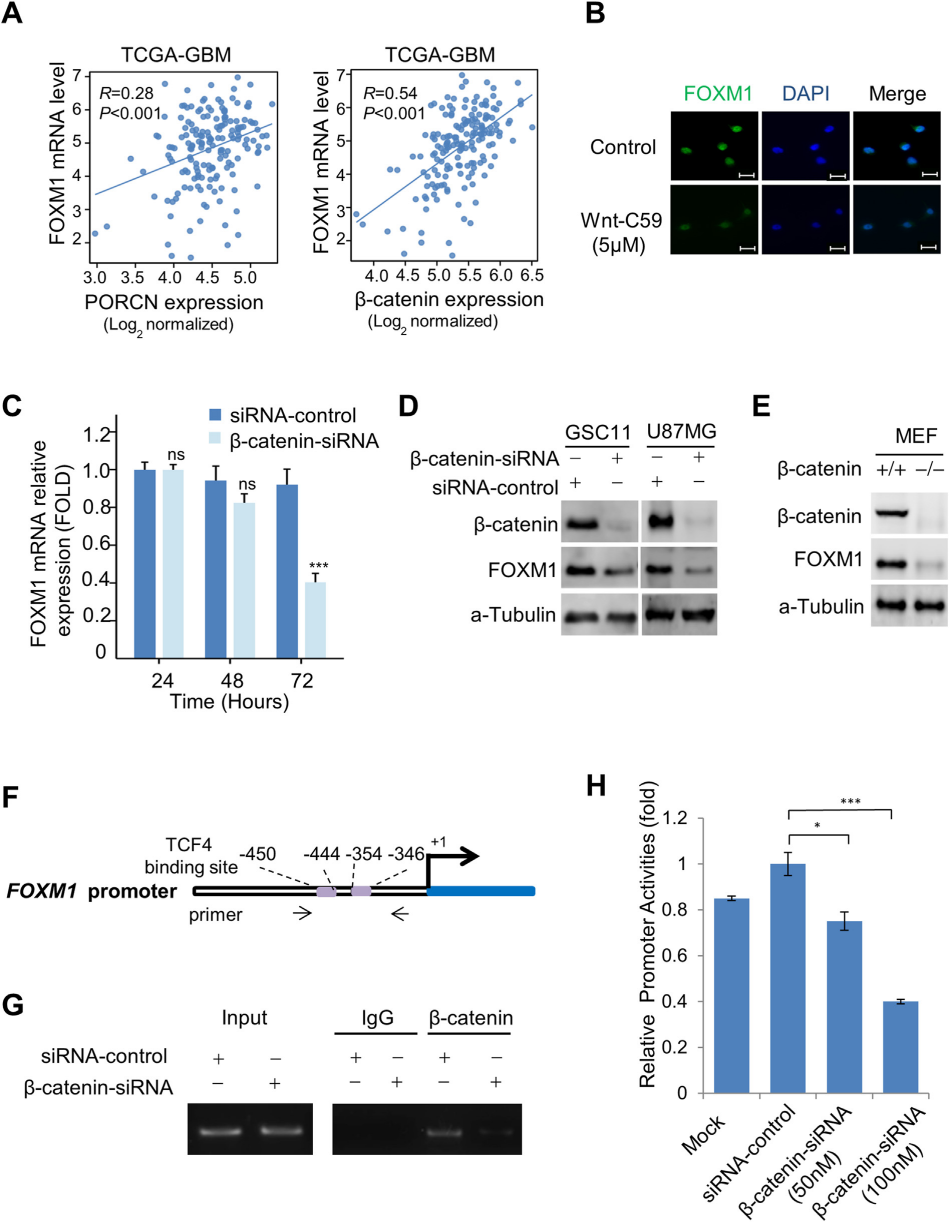
β-catenin interacts with the forkhead box M1 (FOXM1) promoter and regulates its expression. **(A)** The correlations of FOXM1 level with porcupine (PORCN) level, and with β-catenin level were analyzed using the TCGA-GBM dataset. **(B)** GSC11 cells were treated with control DMSO or Wnt-C59 (5 μM) for 24 h. Expression levels of FOXM1 in the cells were analyzed by double immunofluorescence staining for FOXM1 and nuclei (DAPI, blue). Scale bar, 25 μm. **(C)** FOXM1 mRNA levels in GSC11 cells following β-catenin-siRNA treatment at different time points were measured using real-time quantitative PCR. The data were mean ± standard error of the mean. ∗∗∗*p* < 0.001. **(D)** Western blotting analysis of β-catenin and FOXM1 expression in GSC11 and U87MG cells following β-catenin-siRNA treatment for 72 h. **(E)** Comparison of β-catenin and FOXM1 expression levels in β-catenin knockout (*β-catenin*^*−/−*^) and wild-type (*β-catenin*^*+/+*^) MEFs using western blotting analysis. **(F)** Identification of two putative transcription factor 4 (TCF4)-binding sites in the FOXM1 promoter through sequence analysis. **(G)** The chromatin immunoprecipitation assays showed reduced binding of β-catenin to TCF4 regions of the FOXM1 promoter in U87MG cells following β-catenin-siRNA treatment for 48 h. **(H)** The luciferase reporter assays showed reduced FOXM1 promoter activity in U87MG cells following β-catenin-siRNA treatment for 48 h. Bars, standard error of the mean. ∗*p* < 0.05 and ∗∗∗*p* < 0.001.

We previously reported that FOXM1 functioned as a downstream component of Wnt signaling and played a crucial role in β-catenin's transcriptional activity in glioma cells.^4^ We further evaluated the impact of Wnt-C59 on FOXM1. Firstly, in the TCGA-GBM database, FOXM1 mRNA expression positively correlated with the mRNA levels of β-catenin and PORCN (Fig. 4A). Secondly, after Wnt-C59 treatment, FOXM1 expression in GSC11 cells was significantly down-regulated (Figure 2, Figure 4 and 4B). Next, we determined the mechanism underlying Wnt-C59 inhibiting FOXM1 expression. Because Wnt signaling regulates gene transcription through β-catenin, we determined whether β-catenin was an upstream regulator of FOXM1 by examining FOXM1 expression in β-catenin-knockdown GBM cells. β-catenin-siRNA did affect the mRNA level of FOXM1 at 72 h (Fig. 4C). Knocking down β-catenin by siRNAs also significantly down-regulated FOXM1 protein levels (Fig. 4D). To ascertain the effect of β-catenin on FOXM1 expression, we examined FOXM1 expression in β-catenin knockout (*β-catenin*^*−/−*^) and wild-type (*β-catenin*^*+/+*^) MEFs. FOXM1 expression level was decreased in *β-catenin*^*−/−*^ MEFs compared with wild-type MEFs (Fig. 4E), indicating that β-catenin plays an important role in regulating FOXM1 expression. To test whether FOXM1 was a transcriptional target of β-catenin, we first searched for the consensus binding sequence of transcription factor 4 (TCF4), the key transcriptional factor of Wnt/β-catenin pathway, in FOXM1 promoter and identified two putative TCF4-binding sites (Fig. 4F). Then we performed chromatin immunoprecipitation assays on U87MG cells after a 48-h treatment with either siRNA-control or β-catenin-siRNA. Both TCF4-binding regions of FOXM1 promoter bound specifically to endogenous β-catenin protein *in vivo* and β-catenin knockdown strikingly inhibited the β-catenin binding to the regions (Fig. 4G). Moreover, knocking down β-catenin in U87MG cells significantly decreased FOXM1 promoter activity (Fig. 4H). Taken together, these results clearly indicate that β-catenin up-regulates FOXM1 expression through binding to the FOXM1 promoter.

### Wnt-C59 has therapeutic efficacy against intracranial GBM and enhances the effect of TMZ

**Figure 5:**
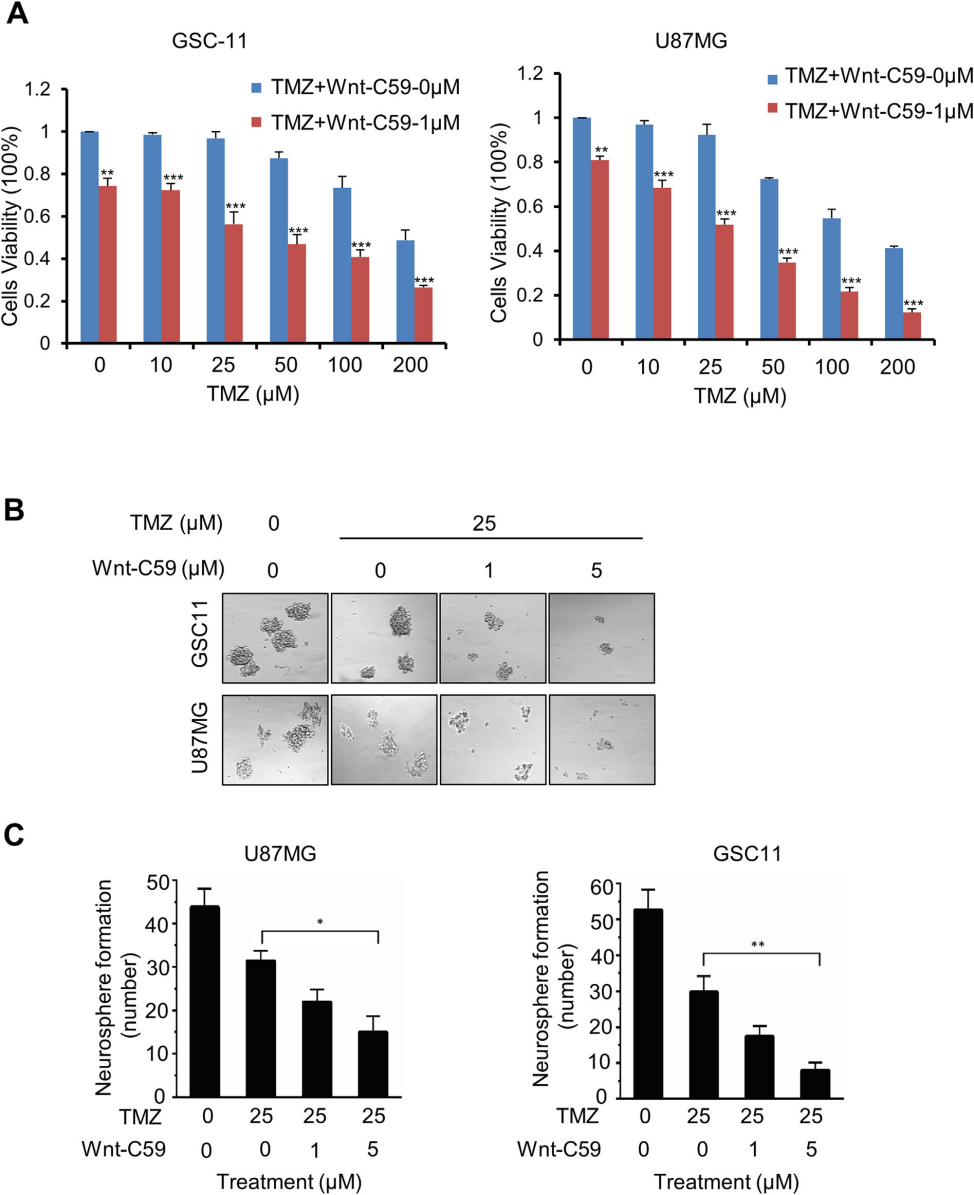
Wnt-C59 sensitizes U87MG and GSC cells to temozolomide (TMZ). **(A)** Proliferation of GSC11 and U87MG cells with or without Wnt-C59 plus TMZ combination treatment was determined by the MTT assay. The values were mean ± standard error of the mean for triplicate samples. ∗∗*p* < 0.01 and ∗∗∗*p* < 0.001. **(B)** Representative images of neurospheres after U87MG and GSC11 cells were treated with Wnt-C59 plus TMZ. **(C)** Neurosphere formation efficiency was assessed in U87MG and GSC11 cells. The neurosphere formation assay was performed by plating dissociated single cells at a density of 1 cell/μL and counting the number of spheres that formed after 7 days. The values were mean ± standard error of the mean for triplicate samples. ∗*p* < 0.05 and ∗∗*p* < 0.01.

Our previous study reported that FOXM1 contributed to TMZ resistance through up-regulation of Rad51.^10^ We sought to determine whether Wnt-C59 sensitized U87MG and GSC11 cells to TMS. Both cell lines were resistant to TMZ treatment especially GSC11, because TMZ could only decrease cell viability at the concentration of 100 μM and above (Fig. 5A). In contrast, combination treatment with 1 μM Wnt-59 and different concentrations of TMZ significantly decreased cell viability as compared with TMZ alone (Fig. 5A). We also observed that Wnt-C59 dose-dependently decreased tumor neurosphere formation efficiency in combination with TMZ (Fig. 5B, C).

**Figure 6:**
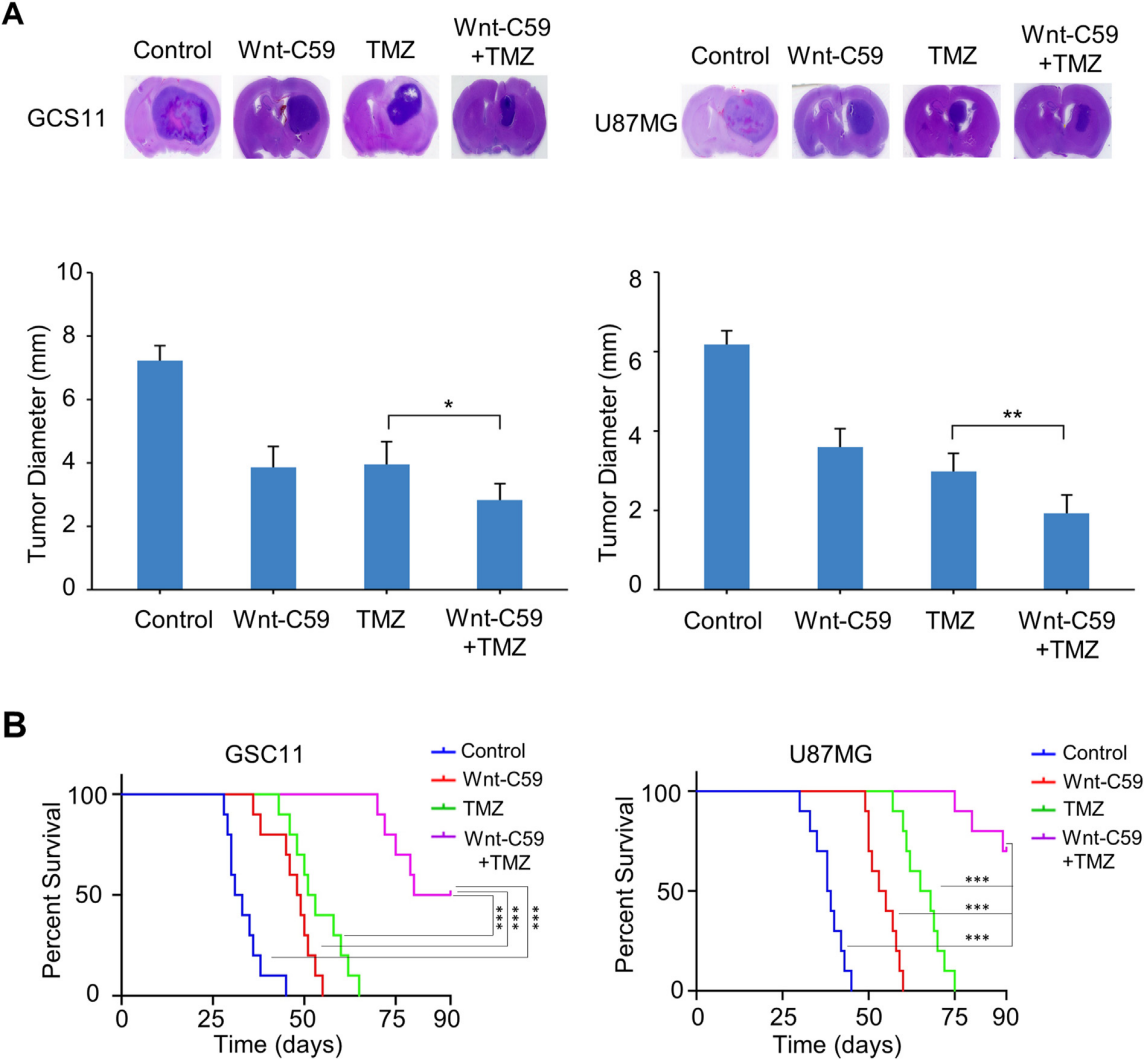
Wnt-C59 in combination with temozolomide (TMZ) inhibits tumor formation and improves mice survival *in vivo*. **(A)** Representative hematoxylin-eosin staining of brains from mice four weeks post tumor implantation. U87MG and GSC11 cells were implanted into the right frontal lobes of nude mice (5 × 10^5^ cells/mouse). TMZ was injected intraperitoneally at a dose of 82.5 mg/kg/d for 7 days, Wnt-C59 treatment was given daily by oral gavage (10 mg/kg/d) for 14 days. Wnt-C59 and TMZ suppressed tumor growth and reduced tumor diameter. Tumor diameters were quantified in hematoxylin-eosin-stained coronal sections by measuring tumor diameters at the largest cross-sectional area. The data were mean ± standard deviation. ∗*p* < 0.05 and ∗∗*p* < 0.01. **(B)** Survival analysis of mice intracranially implanted with GSC11 and U87MG cells. Athymic mice were intracranially implanted with GSC11 or U87MG cells (5 × 10^5^ cells/mouse). Two weeks post-injection, mice were divided into four treatment groups (10 mice per group): i) control, oral saline for 14 days; ii) Wnt-C59, oral gavage (10 mg/kg/d) for 14 days; iii) TMZ, 82.5 mg/kg/d for 7 days; iv) combination of Wnt-C59 and TMZ. Combination therapy significantly increased the survival of GSC11- and U87MG-bearing animals compared with the control group and monotherapy groups. ∗∗∗*p* < 0.001.

Previous studies have suggested that molecules with low molecular weight, small polar surface area, fewer hydrogen bonds, and high lipid solubility are more likely to penetrate the blood-brain barrier.^25^, ^26^, ^27^, ^28^ Based on the physicochemical properties of Wnt-C59 obtained from PubChem, our analyses indicates that Wnt-C59 meets the criteria for crossing the blood-brain barrier (Table S1). Then we sought to assess the therapeutic efficacy of Wnt-C59 on GBM tumor growth using orthotopic GBM models. Compared with vehicle alone, either TMZ or Wnt-C59 alone reduced the tumor diameter, and Wnt-C59 plus TMZ almost abrogated tumor formation (Fig. 6A). In another set of experiments, we assessed the effects of Wnt-C59 alone, TMZ alone, and Wnt-C59 plus TMZ on the survival of U87MG- or GSC11-bearing mice over a 90-day period. As shown in Figure 6B, U87MG- and GSC11-bearing mice became moribund between 28 and 45 days after cell injection. As a monotherapy, Wnt-C59 had a modest effect on prolonging survival of U87MG-bearing mice (median survival: 38.5 days in control group versus 54 days in the treatment group), as well as of GSC11-bearing mice (median survival: 32 days in the control group versus 48.5 days in the treatment group). Similarly, TMZ alone also had a modest effect on prolonging survival of U87MG-bearing mice (median survival: 38.5 days in the control group versus 66.5 days in the treatment group), as well as of GSC11-bearing mice (median survival: 32 days control group versus 52 days in the treatment group) (Fig. 6B). In contrast, the combination of TMZ and Wnt-C59 significantly extended the overall survival of U87MG-bearing mice (70% mice alive in the combinatorial treatment group, *p* < 0.0001), as well as of GSC11-bearing mice (50% mice alive in the combinatorial treatment group, *p* < 0.0001) (Fig. 6B). Taken together, these results indicate that Wnt-C59 sensitizes GBM cells to TMZ and support the clinical application of this combinatorial targeted therapy for GBM.

### Effects of Wnt-C59 treatment on the expression of β-catenin and FOXM1 and their downstream target genes *in vivo*

**Figure 7:**
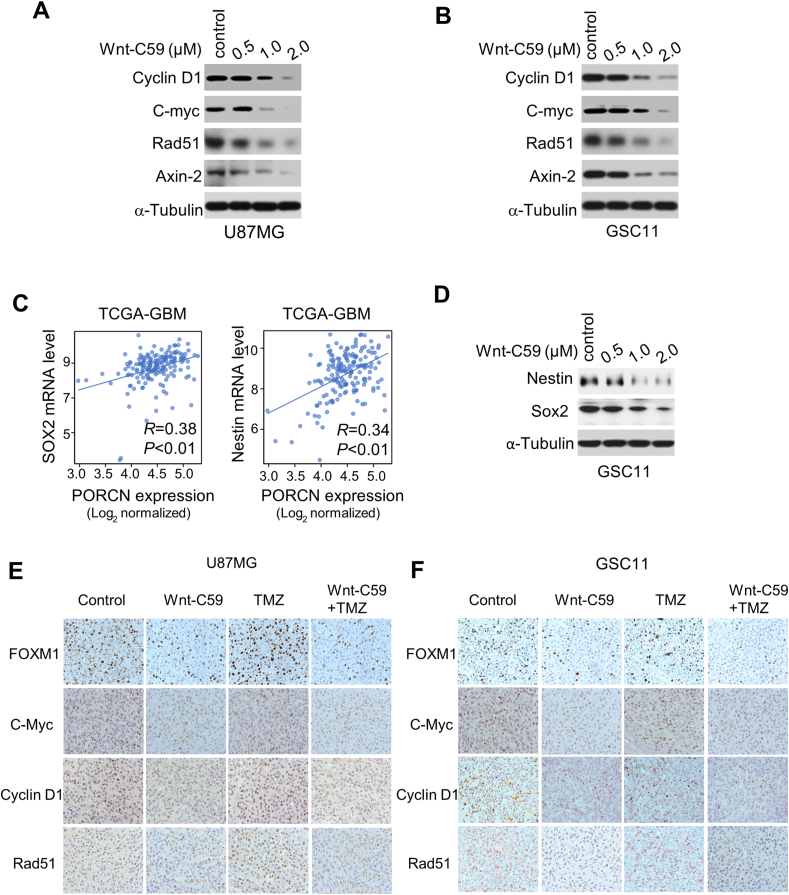
Wnt-C59 inhibits expression of β-catenin's downstream target genes. **(A, B)** Wnt-C59 inhibited the protein expression of β-catenin's and forkhead box M1 (FOXM1)'s downstream target genes in glioma cells. Total levels of cyclin D1, c-Myc, Axin-2, and Rad 51 in U87MG (A) and GSC11 (B) cells treated with 0–2 μM Wnt-C59 were analyzed by western blotting. **(C)** The correlation between the levels of Nestin or SOX2 and porcupine (PORCN) level was analyzed using the TCGA-GBM dataset. **(D)** Wnt-C59 inhibited the expression levels of stem cell markers (Nestin, Sox2). Total levels of Nestin and Sox2 in GSC11 cells treated with 0–2 μM Wnt-C59 for 48 h were analyzed by western blotting. **(E, F)** Representative immunohistochemistry staining of FOXM1, C-MYC, cyclin D1, and Rad51 in U87MG (E) and GSC11 (F) tumors.

To investigate the mechanism by which Wnt-C59 reduced brain tumor formation, we evaluated the effect of Wnt-C59 on the expression of β-catenin downstream target genes, as well as stem cell markers in GSC11 and U87MG cells. Wnt-C59 treatment reduced the protein levels of Wnt/β-catenin signaling downstream targets including cyclin D1, c-Myc, and Axin-2 in a dose-dependent manner. Besides, Rad51, which promotes gliomagenesis and is regulated by FOXM1, was also down-regulated by Wnt-C59 treatment (Fig. 7A, B). Stem cell markers, including Nestin and Sox2, were positively correlated with PORCN levels (Fig. 7C). They were also suppressed by Wnt-C59 treatment (Fig. 7D). Immunohistochemical experiments validated our finding *in vivo* (Fig. 7E, F). These results indicate that Wnt-C59 treatment could inhibit the tumorigenesis of GBM.

## Discussion

GBM is an extremely aggressive tumor with a poor prognosis. Despite advances in neurosurgery, chemotherapy, and radiotherapy, it remains treatment-resistant and inevitably recurs.^29^ Targeting key oncogenic pathways presents promising therapeutic potential and is actively being explored in clinical trials.^30^, ^31^, ^32^, ^33^ Numerous evidence supports the role of the Wnt/β-catenin signaling pathway in gliomagenesis.^34^^,^^35^ Zhang et al demonstrated that FOXM1 is a critical regulator of the Wnt/β-catenin signaling as it mediates β-catenin nuclear accumulation in tumor cells.^4^ FOXM1 expression in human glioma tissue correlates with tumor grade and correlates inversely with patient survival. Furthermore, expression of FOXM1 has been shown to be significantly elevated in recurrent GBM, and targeting FOXM1 sensitized resistant cells to TMZ treatment.^10^ These data indicate that targeting the FOXM1-Wnt/β-catenin signaling pathway could be advantageous for GBM treatment. Wnt-C59 is a small molecule Wnt inhibitor that has shown efficacy in breast tumor models and nasopharyngeal carcinoma.^14^^,^^36^ However, its efficacy in the treatment of GBM has not been investigated. In this study, we demonstrated for the first time the efficacy of Wnt-C59 on an experimental glioma model.

We have found that Wnt signaling is more active in gliomas compared with non-tumor regions, and LRP6 is also phosphorylated in GBM and GSC cells. PORCN, a membrane bound O-acyltransferase family member, is essential for Wnt palmitoylation and secretion, indicating its key role in Wnt signaling.^11^^,^^12^ Our examination of the TCGA-GBM dataset indicates that PORCN is highly expressed in GBM tissues, with elevated levels correlating to a poorer prognosis. We found that Wnt-C59 effectively inhibited LRP6 activation and Wnt signaling in GBM and GSC cells in a dose-dependent manner. We also examined the effect of Wnt-C59 treatment on FOXM1 expression in GBM and GSC cell lines and found that FOXM1 expression decreased in the presence of Wnt-C59 both *in vitro* and *in vivo*. In mice administered with Wnt-C59, the expression of FOXM1 and β-catenin significantly decreased. Furthermore, the expression of Wnt downstream targets genes, cyclin D and c-Myc, were also reduced by Wnt-C59 treatment in GBM cells and GSCs.

We observed that targeting the Wnt/β-catenin pathway with Wnt-C59 compromised the tumorigenicity of GBM cells and GSCs *in vitro* and *in vivo*. Specifically, Wnt-C59 inhibited cell proliferation and invasion of GBM and GSC cells and suppressed the self-renewal of GSC cells. Wnt-C59 monotherapy inhibited tumor growth of GBM cells and GSCs *in vivo* and this effect was significantly augmented with combination treatment with TMZ. Furthermore, the Wnt-C59 and TMZ combination treatment significantly prolonged the survival of the mice bearing GBM and GSC tumors, suggesting that Wnt-C59 may enhance the responsiveness of GBM cells and GSCs to TMZ. This finding supports the potential clinical implementation of this targeted combination therapy for treating GBM.

In summary, our findings demonstrated that targeting the Wnt/β-catenin pathway is a potentially effective avenue for the treatment of GBM. Additionally, Wnt-C59, a small molecular inhibitor of Wnt signaling, effectively reduced the tumorigenicity of GBM *in vitro* and *in vivo*. Due to its oral bioavailability, this treatment was easily combined with standard TMZ therapy and demonstrated a synergistic effect, which offers practical clinical benefits in the treatment of this challenging disease.

## CRediT authorship contribution statement

**Xiaowei Zhang:** Writing – review & editing, Writing – original draft, Visualization, Methodology, Investigation, Formal analysis, Data curation, Conceptualization. **Zhongyong Wang:** Writing – review & editing, Writing – original draft, Visualization, Methodology, Investigation, Formal analysis, Data curation, Conceptualization. **Taohui Ouyang:** Writing – review & editing, Formal analysis, Data curation. **Brayden Wang:** Writing – review & editing, Data curation. **Richard I. Joh:** Writing – review & editing, Data curation. **Suyun Huang:** Writing – review & editing, Writing – original draft, Supervision, Methodology, Investigation, Funding acquisition, Formal analysis, Data curation, Conceptualization.

## Data availability

The datasets used and/or analyzed during this study are available from the corresponding author upon reasonable request.

## Conflict of interests

The authors declare that they have no known competing financial interests or personal relationships that could have appeared to influence the work reported in this paper.
