# Supplementary material for: Targeting Wnt/β-catenin activation in combination with temozolomide leads to glioblastoma inhibition and long-term survival in mice
Source: Genes Dis. 2025 Apr 4;13(1):101624. doi: 10.1016/j.gendis.2025.101624 (PMC12552954; doi:10.1016/j.gendis.2025.101624)
Supplement: Multimedia component 1 [file mmc1.docx]

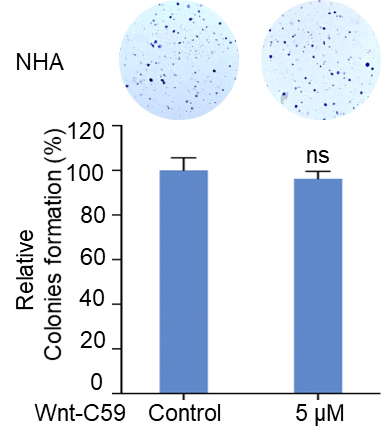


Supplementary Figure 1. Colony formation of NHA cells treated with DMSO (control) or Wnt-C59 (5µM). Representative images of are shown with and without Wnt-C59 treatment. Data are mean ± *SEM* for triplicate samples.

Table S1. Evaluation of the ability of Wnt-C59 to cross the blood-brain barrier.

|  | Requirements | Wnt-C59 |
| --- | --- | --- |
| Polar surface area | <70Å² | 54.9Å² |
| Molecular weight | <450 g/mol | 379.5 g/mol |
| N+O | <5 | 4 |
| Hydrogen bond | <5 donor +10 acceptor | 1 donor+3 acceptor |
| Lipid solubility | ClogP-(N+O)>0 | 4.2-4>0 |
